# Supplementary material for: Resilience of three-dimensional sinusoidal networks in liver tissue
Source: PLoS Comput Biol. 2020 Jun 29;16(6):e1007965. doi: 10.1371/journal.pcbi.1007965 (PMC7351228; doi:10.1371/journal.pcbi.1007965)
Supplement: S1 Appendix — The supporting appendix contains details on simulation and data analysis methods, as well as a minimal model of an adaptive network as discussed in the main text. (PDF) [file pcbi.1007965.s003.pdf]

S1 Appendix

Supporting information for

Karschau et al.: *Resilience of three-dimensional sinusoidal networks in liver tissue.*
PLoS Comp. Biol. 2020.

S1.1 Network permeability computation biased for small sample volumes.

Network permeability is an intensive measure that should be independent of network size for statistically homogeneous networks. This property is the basis for effective medium theories. In reality, however, small sample volumes can introduce a systematic bias in the computation of network permeabilities, see Fig S1. This provides additional motivation of our use of large network samples, as well as for our network generation algorithm to simulate spatially unrestricted networks with prescribed statistical properties.

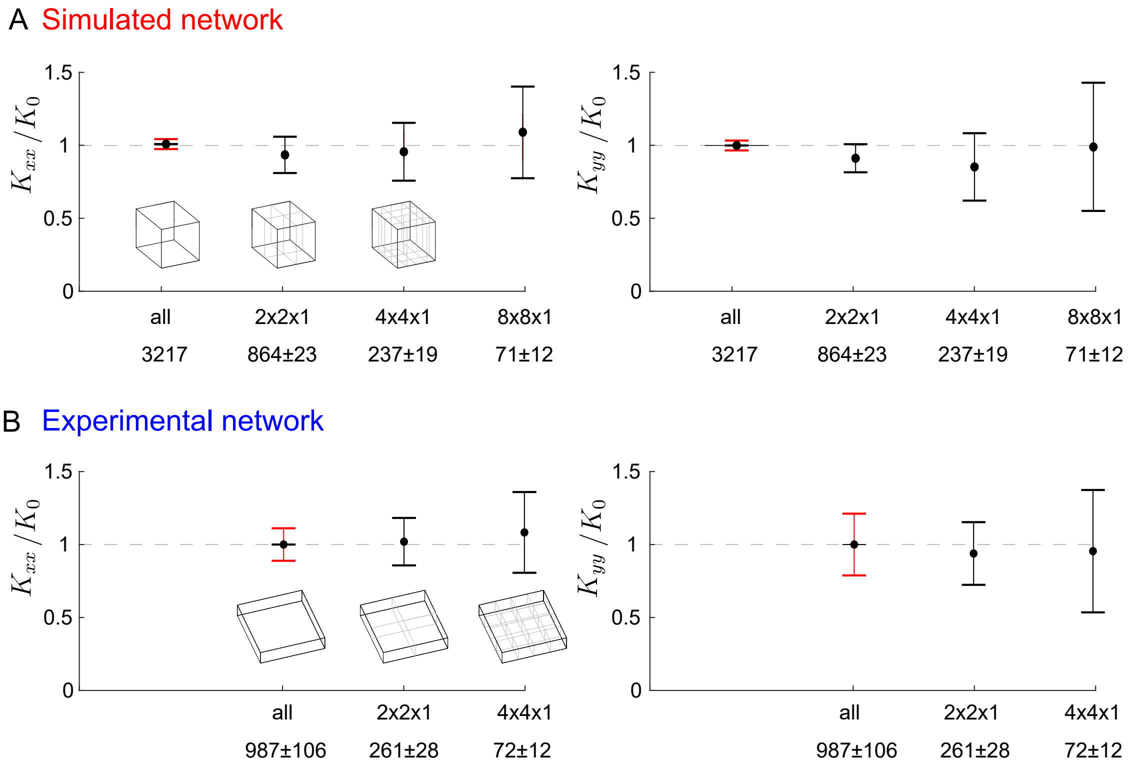

Figure S1. Effect of sample volume on computed network permeability. A. Errorbars show variability of computed permeability of simulated networks for entire sample volume ($1 \times 1 \times 1$, $n = 3$ simulated networks), sample volume divided in 4 equal volumes ($2 \times 2 \times 1$, $m = 4$ non-overlapping sub-samples for each of n full simulated networks), sample volume divided in 8 equal volumes ($4 \times 4 \times 1$, $m = 16$), sample volume divided in 64 equal volumes ($8 \times 8 \times 1$, $m = 64$). For the entire sample volume, errorbars (red) denote the coefficient of variation of permeabilities of the n full networks (standard deviation normalized by mean). For the sub-sample volumes, errorbars (black) denote the standard deviation of the pooled permeabilities of sub-samples ($n \cdot m$), where each permeability was normalized by the permeability of the corresponding full networks; *left:* permeability K_{xx} along direction of nematic alignment, *right:* permeability K_{yy} along y axis perpendicular to direction of nematic alignment. Number of edges is indicated below horizontal axes (mean±s.e.). **B.** Same as panel A, but for sinusoidal networks: for entire sample volume ($1 \times 1 \times 1$, $n = 3$ biological networks), sample volume divided in 4 equal volumes ($2 \times 2 \times 1$, $m = 4$ non-overlapping sub-samples for each of n biological networks), sample volume divided in 8 equal volumes ($4 \times 4 \times 1$, $m = 16$).

S1.2 Permeability-at-risk if low-current edges are removed.

We repeated the analysis of Fig 4C in the main text, but additionally removed low-current edges. If the networks were not perturbed otherwise, we find that their permeability almost did not change. However, if we remove a fraction γ of edges in

addition to these low-current edges, the permeability is substantially reduced, see Fig S2. This shows that low-current edges partially contribute to the resilience of networks, allowing for limited re-routing of flow if high-current edges are removed.

S1.3 Minimal model of metabolic uptake.

We consider a minimal model of metabolic uptake from the sinusoidal network by surrounding tissue (for refined models, see, e.g., Ref.[1]). Let $c(\mathbf{q},t)$ be the concentration of a molecule in solution in the flowing blood at time t at position $\mathbf{q}$ in the sinusoidal network. The dynamics of the concentration profile $c(\mathbf{q},t)$ in the network is governed by diffusion, convection, and uptake

$$\frac{d}{dt}c(x,t) = -\nabla\cdot(\mathbf{v}c) + D\nabla^2 c - \beta c \quad . \tag{S1}$$

Here, we assumed for simplicity a constant uptake rate β with units of an inverse time. Additionally, we assume that concentration is homogeneous across the cross-section of a sinusoid with constant area A. Thus, we need to consider only concentration profiles of a one-dimensional coordinate along a sinusoid edge. Correspondingly, the flow velocity $\mathbf{v}$ for an edge with unit vector $\mathbf{e}$ can be taken as $\mathbf{v} = I/A\mathbf{e}$, where I is the current through the edge.

For efficient computation on a network, we employ an upwind Euler scheme with concentrations evaluated at the node positions $\mathbf{q}_j$ with fixed time step Δt

$$c(\mathbf{q}_j,t+\Delta t) = -\sum_k \max\{0,I_{k,j}\}\,[c(\mathbf{q}_k,t)-c(\mathbf{q}_j,t)]\,\frac{\Delta t}{V_j} + \sum_k D\,[c(\mathbf{q}_k,t)-c(\mathbf{q}_j,t)]\,\frac{A\Delta t}{l_{j,k}V_j} - \beta c(\mathbf{q}_j,t). \tag{S2}$$

Here, the sum $\sum_k$ extends over all nodes connected to node j, with $l_{j,k}$ denoting the length of the edge connecting both nodes. For each node j, we have introduced an *associated volume* $V_j = \sum_k l_{j,k}A/2$. Note that due to the Kirchhoff equation, Eq (8) in the main text, these update rules conserve total mass $\sum c(\mathbf{q}_j)V_j$ if $\beta = 0$. To increase the spatial resolution, we divided each edge of the original network (see Fig 3 in the main text) into $n = 4$ equal parts. For nodes j with small V_j, Eq (S2) is modified by directly using the analytical solution for diffusive exchange between a pair of neighboring nodes, which speeds up computations by allowing to choose larger time steps Δt.

Fig S3 shows steady-state concentration profiles for the network flow from Fig 3D in the main text. Remarkably, the spatial concentration profile varies homogeneously, despite the rather inhomogeneous distribution of flows in the network. Concentration decays approximately exponential along the flow direction; the decay length $\delta \sim v/\beta$ is set by the ratio of a typical flow speed v and uptake rate β. Accounting for diffusion with $D > 0$, homogenizes the concentration profile even further, see Fig S3B.

S1.4 Minimal model for adaptive edge weights.

We consider a minimal transport model with adaptive weights for individual edges: the permeability κ_j of edge j shall be dynamically up-regulated if the current $|I_j|$ through this edge is below a set point I^*, and down-regulated if $|I_j|$ is above this set point

$$\dot{\kappa}_j = \begin{cases} -\rho\,\kappa_0(I^* - |I_j|) & \text{for } 0.25 \le \kappa/\kappa_0 \le 4 \\ 0 & \text{else} \end{cases} \quad . \tag{S3}$$

Here, we included a regularization depending on κ/κ_0, which ensures that edge permeabilities can change at most by a factor 4 (corresponding to a radius change of about $\pm 35\%$ according to Poiseuille's law). Note that the normalized permeability K_{xx} shown in Fig S4 is independent of κ_0.

We find that this local adaptation rule can indeed homogenize the distribution of currents in the network, see Fig S4. However, this minimal model cannot alleviate the structural vulnerability to the removal of high-current edges. These results are insensitive to the choice of set point I^*, provided I^* falls in the range of typical currents of the original network.

S1.5 Permeability-at-risk as function of nematic order.

To gain additional insight into the dependence of both network permeability and permeability-at-risk on the nematic order of a network, we investigated a minimal model of disordered networks, corresponding to random sub-lattices of a cubic lattice with prescribed nematic order parameter, see Fig S5. Generally, we observe that nematic order increases both network permeability and permeability-at-risk if the total length of the network is kept fixed. In contrast, adding redundant edges perpendicular to the mean flow direction, increases network resilience for a given number of removed edges. Simulations for cubic lattices of different sizes ($20\times20\times20$, $150\times10\times10$) gave similar results.

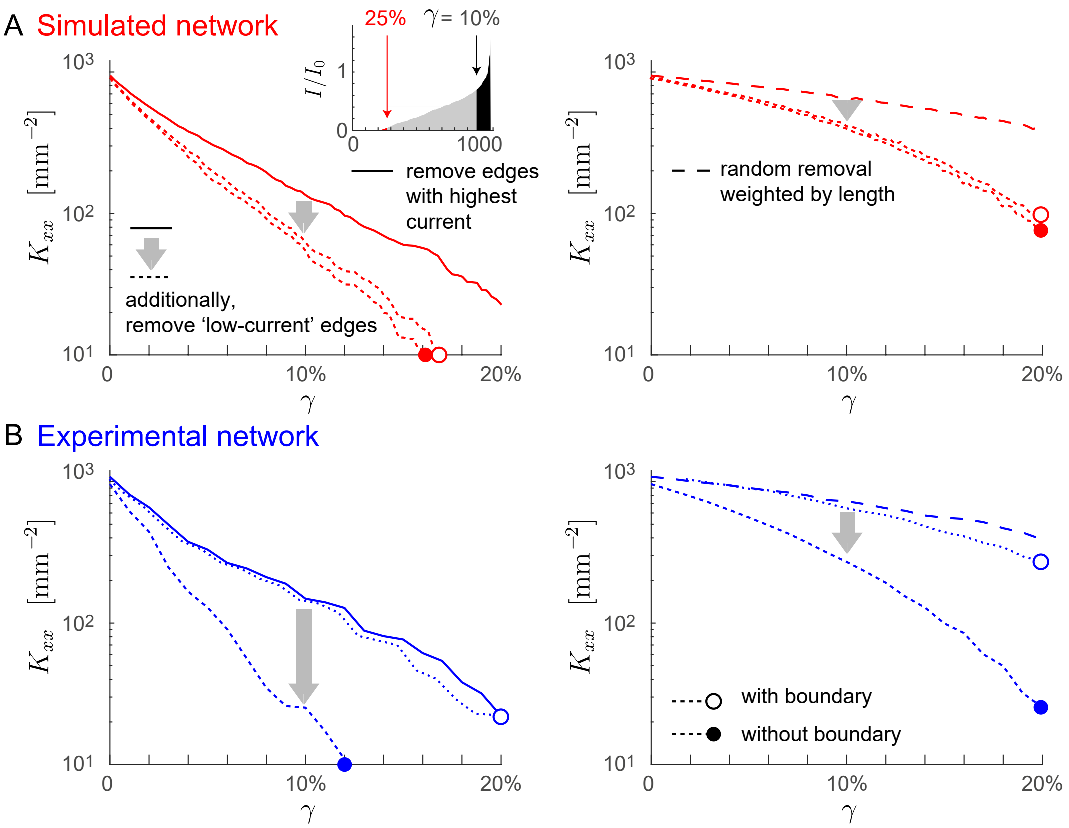

Figure S2. **Low-current edges contribute to resilience.** We repeated the analysis of Fig 4C in the main text, but additionally removed low-current edges from both simulated and sinusoidal networks. **A.** *Left:* We show again the permeability-at-risk curve for simulated networks from Fig 4C (solid red, scenario i; $\lambda = 0.2$, mean of $n = 3$ simulated networks), where a fraction γ of edges is removed, starting with the edges that carry the highest current in the unperturbed network. We compare this curve to a second permeability-at-risk curve, where additionally 25% of the edges that carry the lowest current in the unperturbed network are removed (dotted, marked with open circle). Alternatively, we can likewise remove 25% of all edges, choosing again those that carry the lowest current in the unperturbed network, yet restrict the selection to edges that do not touch the boundary of the simulation domain (dotted, marked with filled circle). The fraction γ is always measured relative to the total number of edges in the unperturbed network. Hence, for the dotted curves, the number of edges of the perturbed networks equals $75\% - \gamma$ of the number of edges in the unperturbed networks. *Right:* Same as left panel, but for random removal of a fraction γ of edges (scenario ii), with removal probability proportional to edge length. *B.* Same as panel A, but for the sinusoidal networks. Of note, the permeability-at-risk curves for the networks with low-current edges removed differ depending on whether we restrict the selection to low-current edges that do not touch the boundary (dotted curve, marked with filled circle), or not (dotted curve, marked with open circle). This can be attributed to the small dimensions of the tissue sample, notably in z direction, which results in a number of edges at the boundary that carry almost zero current.

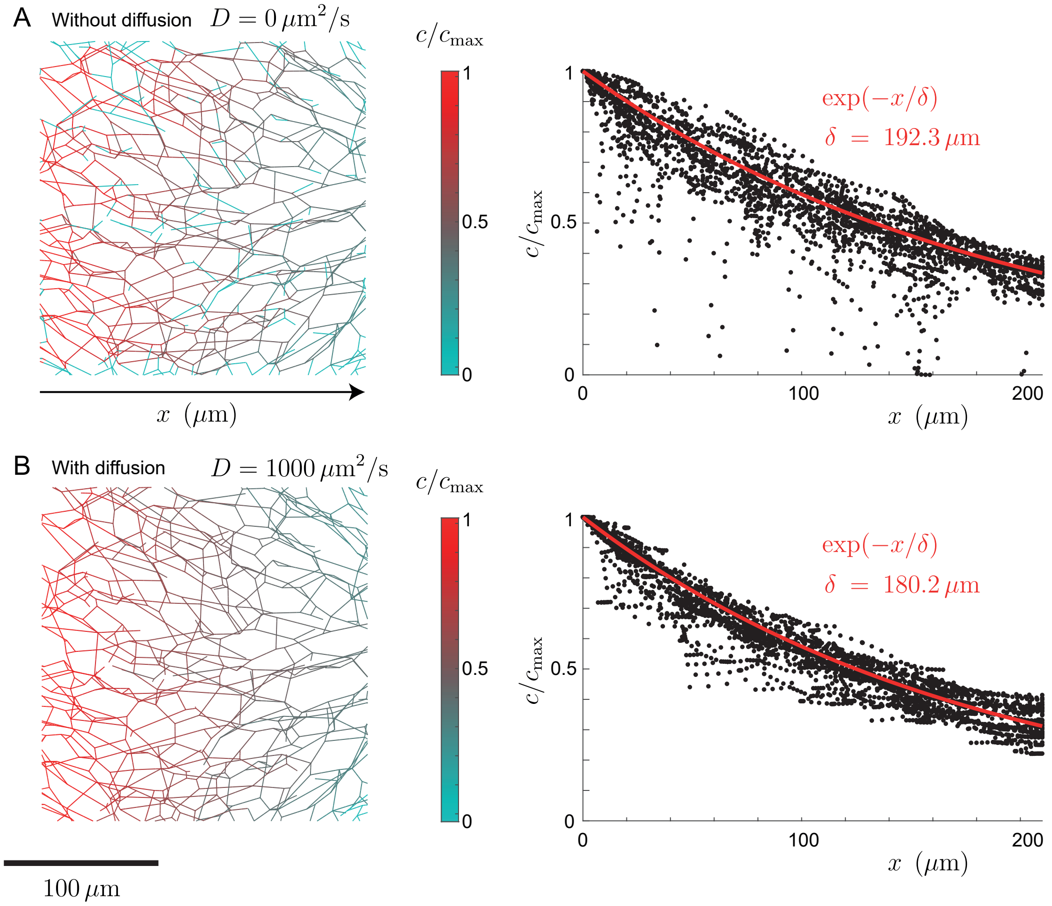

Figure S3. Computed concentration profile of a prototypical metabolite using a minimal model of metabolic uptake from the sinusoidal network. A. Steady-state concentration profile computed according to Eq (S2) for a case without diffusion. Approximately, the concentration profile decays exponentially along the pressure gradient direction (black: steady-state concentration $c(\mathbf{q}_j)$ normalized by maximal concentration $c_{\max}$ as function of position $x = \mathbf{q}_j \cdot \mathbf{e}_x$, red: fit $\exp(-x/\delta)$). **B.** Same as panel A, but accounting for diffusion. Parameters: uptake rate $\beta = 0.1\,\mathrm{s}^{-1}$, diffusion coefficient $D = 0$ in A, $D = 1000\,\mu\mathrm{m}^2/\mathrm{s}$ ($\sim$ diffusion coefficient of oxygen) in B; for flow computation: sinusoid radius $R = 3\,\mu$m, sinusoid cross-sectional area $A = \pi R^2$, dynamic viscosity of blood $\mu = 3.5 \cdot 10^{-3}\,\mathrm{Pa\,s}$[2], resistance per unit length $\kappa = 8\mu/(\pi R^4)$ (assuming Poiseuille flow), pressure difference $\Delta p = \Delta p_0 L_x / L_0$ with pressure difference between PV and CV $\Delta p_0 = 100\,\mathrm{Pa}$[3,4], typical distance between PV and CV $L_0 = 500\,\mu$m, spatial dimension of region of interest $L_x = 210\,\mu$m.

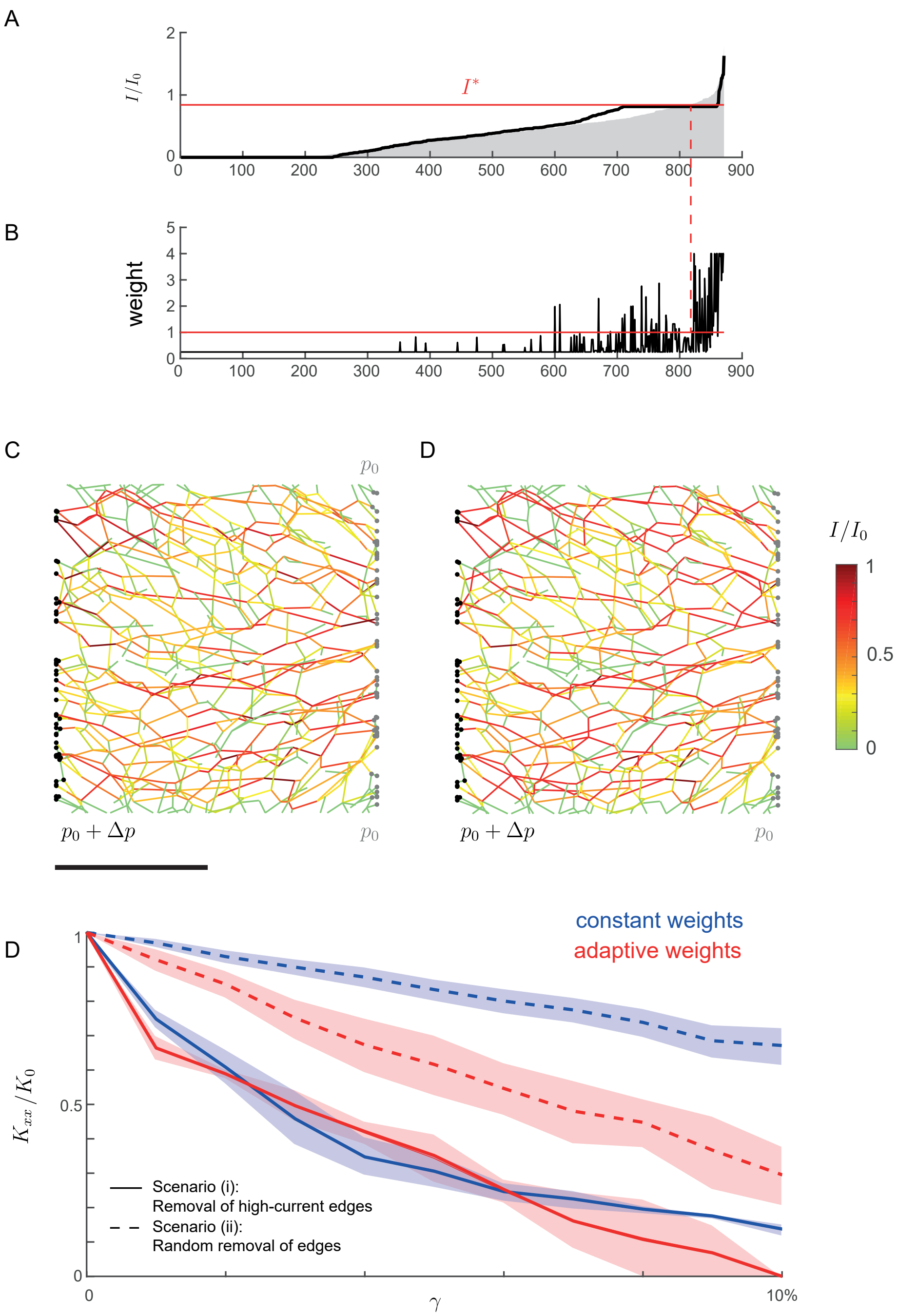

Figure S4. Minimal model for adaptive edge weights. A. Distribution of individual currents along network edges after dynamic adaptation of edge permeabilities according to Eq (S3) (black), compared to those for the original network (red). **B.** Corresponding weights $\kappa_j/kappa_0$ for each network edge. **C.** Spatial distribution of currents both for the case of constant weights (left, identical to Fig 3D in the main text, and the new case of adaptive weights (right). **D.** Normalized permeability-at-risk $K_{xx}(\gamma)/K_xx(\gamma=0)$ as function of fraction γ of removed edges for both the case of constant weights (blue, mean±s.e., see also Fig 4A in the main text), and the new case of adaptive weights (red), for both scenario (i) (removal of high-current edges, solid) and scenario (ii) (random removal of edges, dashed). We used the experimental network from Fig 4A; results for the other experimental networks as well as simulated networks are similar. Parameters: $I^* = 0.83\,I_0$, corresponding to the 95% percentile of the distribution of currents in the unperturbed network; a steady-state had been reached for the chosen simulation time $t_{\max} = 10^4/\rho$.

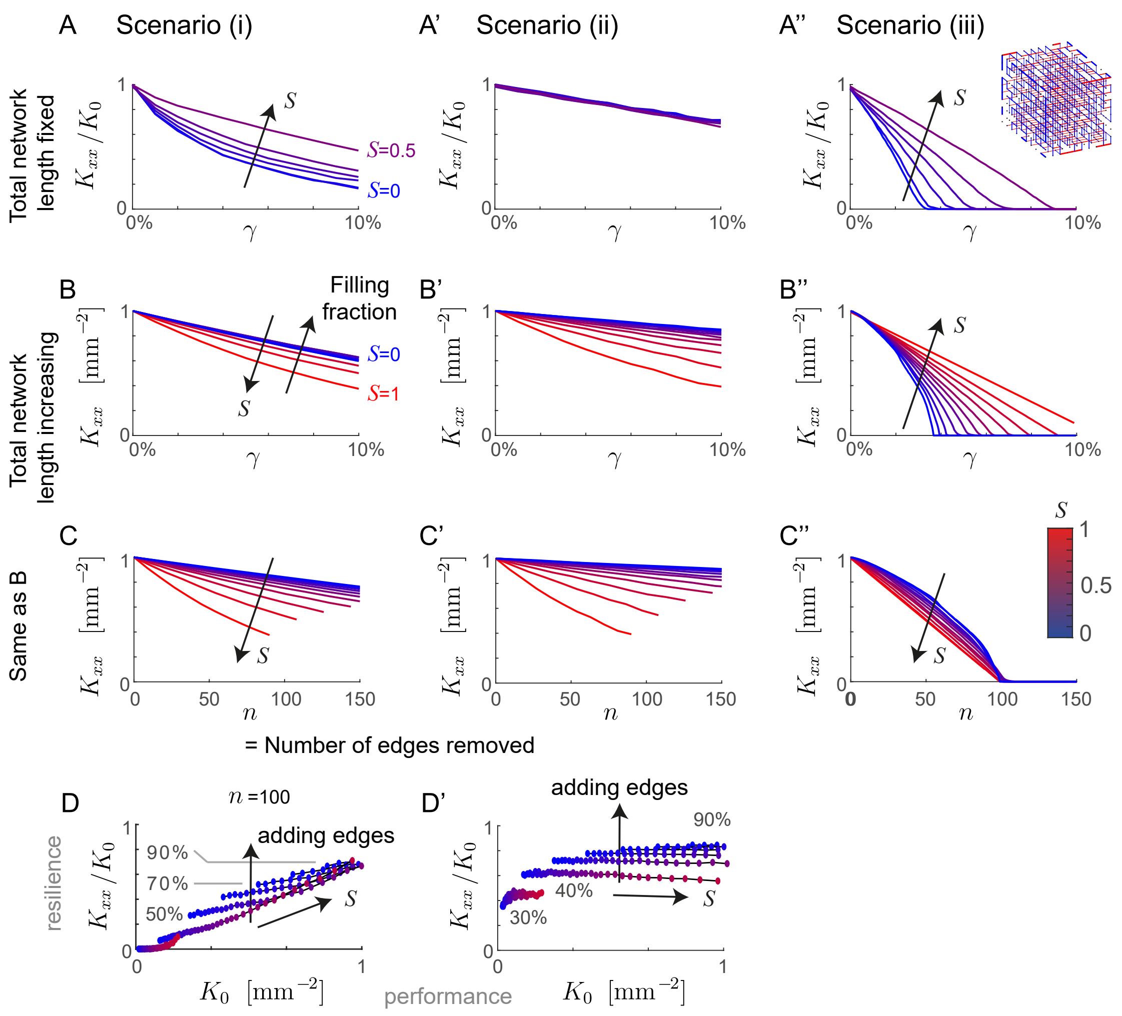

Figure S5. Nematic order increases both network permeability and permeability-at-risk. A. Relative change in permeability as function of the fraction γ of removed edges for a minimal model of disordered networks with different nematic order parameters S for scenario *i* (removal of high-current edges). Specifically, we used random sub-lattices of regular cubic network of dimension $10 \times 10 \times 10$ with filling fraction 42 % and prescribed nematic order parameter S, where the case $S = 0$ was considered in Fig 4C in the main text (cyan). Permeabilities $K_{xx}(\gamma)$ along the direction of nematic order were normalized by the permeability $K_0 = K_{xx}(\gamma = 0)$ in the absence of a perturbation. **A'.** Same as panel A, but for scenario *ii* (random removal of edges). **A''.** Additionally, we considered a third scenario *iii*, for which edges were removed one-by-one, always selecting the edge with the highest current at each step. This scenario *iii* corresponds to a case of directed percolation. **B-B''.** Same as panels A-A'', but now using sub-lattices of variable filling fraction. Specifically, the sub-lattices contain all possible edges parallel to the x direction, as well as a random selection of edges in a perpendicular direction, thus corresponding to a variable filling fraction ranging between 33 % ($S = 1$) and 100 % ($S = 0$). **C-C''.** Same as panels B-B'', but for a different horizontal axis that now shows the total number n of removed edges (instead of the relative fraction γ as in panels B-B''). **D.** We consider a two-dimensional space of objectives, with permeability K_0 in the absence of a perturbation on the horizontal axis (performance), and the relative change in permeability K_{xx}/K_0 after a perturbation on the vertical axis (resilience). All permeabilities are computed along the direction of nematic order; the perturbation consists of a removal of $n = 100$ high-current edges, corresponding to scenario *i*. Each dot indicates a random sub-lattice of a cubic $10 \times 10\times$ lattice with prescribed nematic order parameter S (color code) and variable filling fraction ranging from 30% to 90% as indicated; dots corresponding to the same filling fraction are connected by a line. We observe that for a fixed filling fraction, i.e., constant total network length, both objectives increase upon increasing S. In contrast, adding redundant edges that do not increase network permeability K_0 increases network resilience K_{xx}/K_0. **D'.** Same as panel D, but for random removal of $n = 100$ edges, corresponding to scenario *ii*. While network permeability K_0 increases as a function of the network's nematic order parameter, network resilience is largely independent. As in panel D, adding redundant edges increases network resilience K_{xx}/K_0. Lattice spacing: 1 mm.

References

1. Meigel, F. J. & Alim, K. Flow rate of transport network controls uniform metabolite supply to tissue. *J Roy. Soc. Int.* **15**, 20180075 (2018).

2. White, D., Coombe, D., Rezania, V. & Tuszynski, J. Building a 3d virtual liver: Methods for simulating blood flow and hepatic clearance on 3d structures. *PloS one* **11**, e0162215 (2016).

3. Debbaut, C. *et al.* Perfusion characteristics of the human hepatic microcirculation based on three-dimensional reconstructions and computational fluid dynamic analysis. *J. Biomech. Engin.* **134**, 011003 (2012).

4. Ricken, T. *et al.* Modeling function–perfusion behavior in liver lobules including tissue, blood, glucose, lactate and glycogen by use of a coupled two-scale pde–ode approach. *Biomech. modeling mechanobiology* **14**, 515–536 (2015).
