# Supplementary material for: Resilience of three-dimensional sinusoidal networks in liver tissue
Source: PLoS Comput Biol. 2020 Jun 29;16(6):e1007965. doi: 10.1371/journal.pcbi.1007965 (PMC7351228; doi:10.1371/journal.pcbi.1007965)
Supplement: Attachment — Submitted filename: karschau_network_resilience_rebuttal.pdf [file pcbi.1007965.s005.pdf]

TECHNISCHE UNIVERSITÄT DRESDEN

Technische Universität Dresden, 01062 Dresden

Editorial Office
PLoS Computational Biology

PD Dr. rer. nat. habil. Benjamin M. Friedrich
Biological Algorithms Group

Tel: +49 351 463 42641
E-Mail: benjamin.m.friedrich@tu-dresden.de
URL: https://www.cfaed.tu-dresden.de/friedrich-home

PCOMPBIOL-D-19-02210 / Resubmission
Karschau et al. Resilience of three-dimensional sinusoidal networks in liver tissue

Dear Prof. Daniel A Beard,
Dear Editorial Staff,

May 4, 2020

Hereby, we resubmit a revised manuscript that addresses all comments from the reviewers.

We summarize the major changes to our manuscript (point-by-point answers to all comments of the referees are included further below):

- We now detail three **benefits of simulated networks**: 1) reduced statistical fluctuations due to increased network size, see Fig. S1; 2) reduced boundary effects due to increased spatial extent in *z* direction, see Fig. S2; 3) possibility to tune the nematic order of simulated networks.
- We now include a **minimal model of an adaptive network** in the SOM text, demonstrating that a simple local adaptation rule can partially homogenize the inhomogeneous distribution of currents in the networks, yet not alleviate the structural vulnerability to the removal of high-current edges.
- We now discuss the **effect of nematic order on permeability-at-risk** using a minimal model in a new supplemental figure S5: increasing nematic order always increases both the permeability in the absence of a perturbation as well as the permeability-at-risk in the presence of a perturbation. The maximal nematic order observed in sinusoidal networks matches the maximal nematic order that we can achieve in simulated networks; this maximal value of the nematic order parameter is set by geometrical constraints imposed by network statistics.

We are confident that the revised manuscript now meets the high standards of PLoS Computational Biology in terms of quality, novelty and general interest.

On behalf of all authors,
Yours sincerely,
Benjamin M Friedrich

We thank both referees for their constructive feedback, and positive evaluation. Referee #1 asserted that our work is "generally well-written", "interesting", and "sound".

Answer to Referee #1

In their article ``Resilience of three-dimensional sinusoidal networks in liver tissue," Karschau and co-workers investigate the structure and topology of sinusoidal networks in the liver. Based on data from their Ref. 13, the authors construct an algorithm for *in-silico* generation of arbitrarily large transport networks with the same statistical properties as their real data. They consider both geometry (position of branch points as quantified through a radial distribution function) and topology (node degree and edge length distribution). In a second step, the authors include nematic alignment into their generation algorithm.

Based on their statistical model, the authors proceed to characterize resilience of their statistical and real networks to removal of edges. They find that while the networks are generically resilient to removal of random edges, the loss of certain high-flow edges can lead to larger damage, as quantified by a loss in overall hydraulic permeability. Furthermore, the authors find that weak nematic alignment of the edges with overall flow direction can increase permeability while retaining robustness.

The paper is generally well-written and interesting. The network generation method appears to be sound in terms of results.

Response:
We thank the reviewer for his/her positive evaluation of our work and address comments below.

However, I have a few concerns and remarks regarding details.

(*) While the authors employ multi-objective optimization techniques to find a Pareto front of trade-offs between their objectives, this Pareto front is then never actually used. In fact, after re-scaling the objectives appropriately, the authors end up simply averaging the individual objectives.

Given that the goal was to reproduce the statistics of their real data, scaling and averaging seems to be the most straightforward and direct fitting method.

I suspect that the normalization involving utopia and nadir points is of relevance here, but why these are chosen should be made more clear. So, while the Pareto terminology is interesting, it seems to me that it might not actually needed to obtain the results in the paper. This is consistent with the authors' observation that their algorithm is robust with respect to choice of alpha.

Response.
We agree.

We find *a posteriori* that the algorithm is robust with respect to the specific choice of weighting factors and that a simple mean of the two (normalized) objective functions suffices. Yet, *a priori*, this was not obvious. Furthermore, the normalization of the objective functions by their respective nadir and utopia points is a crucial step. As a technical point, we note that the nadir points cannot be computed by simply optimizing a second objective as the problem exhibits multiple optimal networks with vastly different values for the first objective. Instead, we need to compute nadir points as limit values of multi-objective optimization, where one of the weights converges to zero, i.e., as the end points of the Pareto front.

Thus, although Figure 2F does not address Pareto optimality with respect to two conflicting functional characteristics, it nonetheless shows that our multi-objective network generation algorithm works robustly and how it does so.

We added the following text in the revised manuscript:

> We thus find *a posteriori* that our algorithm is robust with respect to the particular choice of α. In the following, we use the value α*=… which corresponds to choosing equal weights for the individual cost functions C_d and C_e after these have been normalized such that their utopia and nadir points are mapped to zero and one, respectively, see Methods section for details. This normalization requires in particular knowledge of the nadir points, which can only be computed as limits of the Pareto front, but not using single-objective optimization alone.

(*) The authors conclude that ``weakly nematic networks optimize performance without compromising resilience." While the authors' results support this statement, here, Pareto methods would appear to be actually the appropriate tool for quantification. In an objective space comprising performance and permeability-at-risk, networks with weak nematic alignment should be on the Pareto front, and those without should not be Pareto efficient.

Response.
This a very interesting point, which we want to clarify.

In our study, we do not observe a trade-off between performance and resilience as function of a network's nematic order parameter. Instead, we find that disordered networks with higher nematic order parameter not only have a higher permeability in the absence of perturbation, but also display a higher permeability-at-risk, provided the total length of the network is kept fixed.

Notwithstanding, adding redundant edges perpendicular to the mean flow direction increases network resilience for a given number of removed edges. We argue that this could be a possible design principle of sinusoidal networks, where total network length is not a severe constraint.

In the revised manuscript, we have added a new paragraph in the 'Discussion' that clarifies this point. Additionally, we added a new Supplemental Figure S5, which illustrates this effect, using a minimal model of disordered networks given by sub-lattices of a regular cubic lattice.

We have reworded the phrase “weakly nematic networks optimize performance without compromising resilience”, which seems to have wrongly suggested that weakly nematic networks represent an optimal trade-off between performance and resilience. Instead, other design requirements of sinusoidal networks (reflected by their node degree and edge length distribution) imply a maximal value of the nematic order parameter, see also Fig. 3C. Interestingly, this maximal value is close to the maximal nematic order parameter of the three sinusoidal networks analyzed.

We apologize if the original wording caused a misunderstanding.

(*) The authors mention that flows in their simulated networks are very inhomogeneous, contrary to intuition. Is it possible that this inhomogeneity is related to their choice of pressure boundary conditions? More specifically, what motivates the choice of fixing pressures instead of flows at the boundaries, and would fixing boundary flows lead to more homogeneous bulk flows?

Response.
As a new control, we performed flow computations with pre-scribed flows at the boundary: we predict an inhomogeneous flow distribution also in this case, see Fig. 1 below.

Note that in the liver, blood flow in the sinusoid network is driven by a pressure difference between the portal and central vein, which motivates a boundary condition with prescribed pressure.

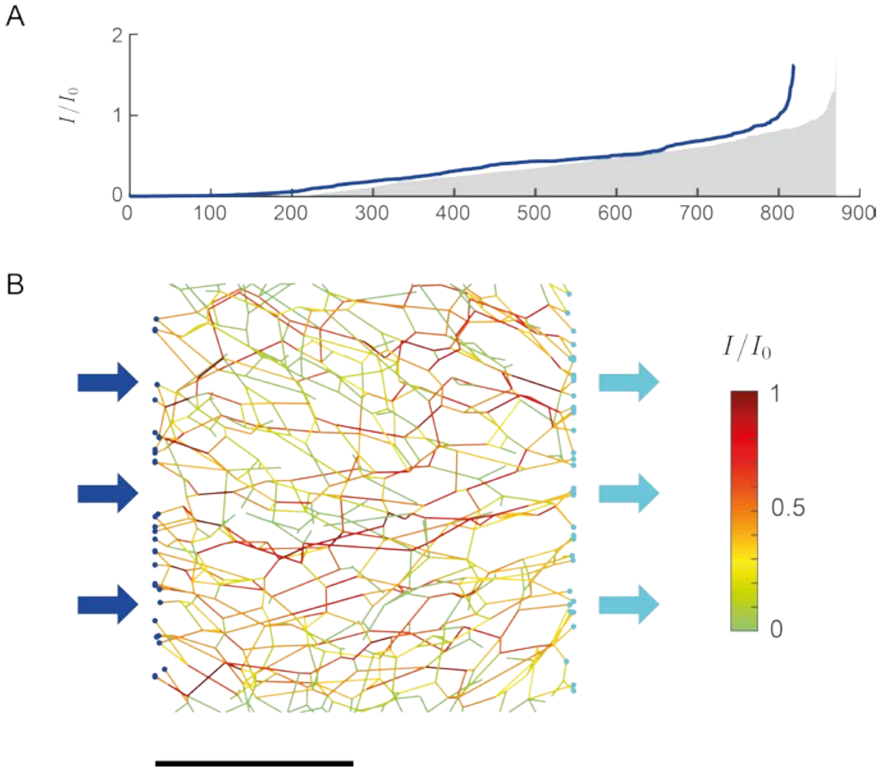

Figure 1. *Computed flow through the sinusoidal network as in Fig. 3D, but for imposed inflow and outflow at boundary nodes on opposing boundary surfaces.* **A.** Distribution of currents for all network edges for the new boundary conditions of prescribed inflow and outflow (blue). For comparison, the distribution of currents for the boundary condition of prescribed pressure difference used in Fig. 3D is shown in gray. **B.** Spatial distribution of flow in the sinusoidal network for the new boundary conditions of prescribed inflow and outflow shown in color code. *Notes on methods:* The prescribed inflow and outflow per edge were chosen to give the same total current as in Fig. 3D. As prescribed inflows and outflows at boundary edges are not necessarily compatible with the Kirchhoff equations, we used linear least-squares optimization to compute the distribution of flows; the resultant mean normalized deviation of computed currents from the prescribed values was ±15%. Reference current I_0 as in Fig. 3D (results are independent of Δp, as this value only sets the scale of the resistance κ per unit length). Note that we removed small disconnected network components prior to the computations, thus the reduced number of network edges in panel A.

(*) While available in Ref. 13, it would be useful to briefly mention again the size of the original data set in terms of both number of samples and size of the networks. How much larger than the real networks are the authors' artificial ones? How large is the statistical advantage of using artificial networks over the real data?

Response.
Following the suggestion of the referee, we now include statistical information on the size of experimental and simulated networks.

The simulation of synthetic networks has three advantages:

1. It allows to analyze larger networks samples. This increases the precision of computed mean network permeabilities.

2. It allows to analyze network volumes of increased spatial extent in *z* direction, thereby reducing boundary effects in flow computations.

3. Using simulated networks, we can tune the nematic order parameter *S* of the network, which is not possible for experimental networks.

We now write in the revised manuscript:

> The simulated networks offer a three-fold advantage compared to the spatially restricted experimental samples: (i) they allow to analyze transport properties in larger networks (here: about 2.6-fold more nodes compared to central regions of interest), thus reducing statistical fluctuations arising from small sample volumes (see Fig. S1), (ii) they allow to analyze networks with larger spatial extent in *z* direction, thus reducing boundary effects in flow simulations (see also Fig. S2), and (iii) they allow to change nematic order of the network by an external control parameter, as considered next.

We also write:

> We analyzed *n*=3 sinusoidal networks in total, each with approximate sampling volume 420 μm x 450 μm x 90 μm and 1643, 2689, 1996 nodes, respectively, which all gave similar results.

(*) The plots in Fig. 1 DEF suggest that the real data does not provide particularly smooth distributions (in particular for the edge lengths). What precautions have been taken to avoid overfitting of statistical fluctuations in the data? How large is the error in the PDFs obtained from the real data?

Response:
The histograms in Fig. 1 DEF use a small bin size; thus, small-number fluctuations are indeed visible. For the simulation of networks, we used a larger bin size, corresponding to a smooth PDF, see Fig. 2 E'D'. This reduces the vulnerability to overfitting.

More importantly, overfitting could occur and be harmful if the simulated networks would re-use the node points of the experimental networks. However, our network generation algorithm computes node positions *de novo*, using only statistical features of the distribution of node positions of experimental networks.

For the convenience of the reviewer, we included below the cumulative distribution function for node degree and edge length for three different sinusoidal networks, which shows the small variability between different data sets, see Figure 2.

CDF

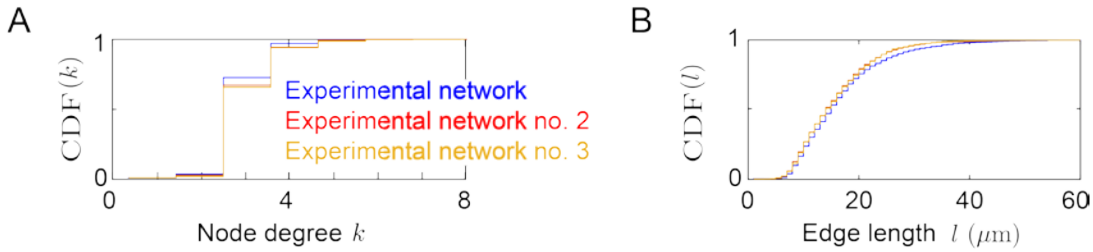

Figure 2. Cumulative distribution functions for different experimental networks. A. Cumulative distribution function of node degree of different sinusoidal networks (*n*=3). **B.** Same as panel A, but for the edge length distribution. Experimental network no. 1 (blue) corresponds to the network shown in Fig. 2C'D' of the manuscript.

As an additional test, we had previously simulated networks using also the PDFs of different experimental samples, which gave very similar results.

We could include such a simulation in a second revision as a Supplemental Figure in the Supporting Online Material if requested by the referee. (We have not yet done so, because we would like to re-rerun these simulations with the latest parameter set as used for the current Figure 2 for consistency. We cannot do so at the moment for very practical reasons as we cannot access our workstation computers while our university operates in emergency mode.)

Minor typos:
- author summary: vene, leafs -> vein, leaves
- p. 10: extend -> extent

We corrected these typos.

Reviewer #2

Summary

This study is motivated by the question "can 3D microvascular networks work if individual links fail?" The authors first quantify the statistical geometry of high-resolution images of mouse hepatic sinusoidal networks. They operate from a graph theory perspective to define the bifurcations as nodes and the blood vessels as edges. They use a Monte-Carlo algorithm to generate synthetic networks, which reproduce the statistical features of mouse hepatic sinusoidal networks. Node degree and edge length are the optimized quantities. The authors also quantify the bulk direction network edges (blood vessels) are oriented by a nematic order parameter which is also included in their multi-objective cost function for Monte-Carlo network generation. A minimal model of blood flow is used to predict the transport properties of the network. The critical assumptions made are that blood vessels have a constant resistance per unit length, and that there is a uniform pressure drop across the simulated cuboid region boundary (pressure drop along x-axis; at x=0 all nodes are set to $p_0 + \Delta p$, and all nodes at x=L nodes are set to p_0). Normalized permeability of the network is computed via Darcy's law. Network resilience is quantified by a new metric called "permeability-at-risk," where normalized permeability is plotted as a function of the fraction of removed edges. Two scenarios are considered for the removal of edges: (i) removal of high current edges; and (ii) removal of random edges. Simulations of these two scenarios are used to address the original question – can microvascular networks work if individual links fail?

Major Findings

- The sinusoidal network is a subgraph of the Delaunay graph (of the bifurcation nodes). Implies that sinusoidal networks reflect design requirements in which every hepatocyte in the tissue is in contact with the vascular network
- The sinusoidal network contains the minimum spanning tree. Implies that sinusoidal network design/growth minimizes path lengths throughout the network
- Nematic order determines the anisotropy of network permeability.
- Removing high flow edges is more detrimental compared to random removal of edges. At a given fraction of edges removed from the network, removing high flow edges makes it more likely for the permeability (in the direction of the pressure drop) to collapse to zero. In other words, low flow edges enable network resilience.

Critiques

- Nematic alignment/order needs to be clearly defined at the beginning of the "Nematic order determines anisotropy of network permeability" section. A quick google search of the term gives a lot of information on liquid crystals in LCD TV displays. By context I was eventually able to infer what it was measuring, but it was not immediately intuitive.

Response.
We followed the suggestion by referee #2, and now explain both in the introduction and the mentioned section the physical concept of 'nematic alignment' and its quantification by a nematic order parameter.
In fact, these concepts have been first introduced to describe liquid crystals (reviewed e.g. in the book by deGennes & Prost), and have later been applied also to living matter, e.g., cytoskeletal structures.

We now write in the introduction:

> Sinusoidal networks display a weak nematic alignment along the direction of flow, i.e., the edges of the network are not oriented isotropically in all space directions, but exhibit a tendency to be aligned towards a common axis.

We write in the section "Nematic order determines anisotropy of network permeability":

> Nematic order describes the partial alignment of local axes, such as direction axes of anisotropic objects. This concept was originally introduced to describe the mutual alignment of elongated molecules in liquid crystals [deGennes et al.], yet has been extended to characterize also mutual alignment of biological structures, such as cytoskeletal filaments, or polarized cells in a tissue. Technically, one distinguishes polar order (parallel alignment of single-headed vectors ↑↑, but not anti-parallel alignment ↑↓) and nematic alignment (parallel alignment of undirected axes, ↕↕). The local mean direction of nematic alignment is called the *nematic director*.

- In equation (5), you should clarify what the angled brackets indicate. Is it an inner product, or a statistical average? My guess is that it's an average, as you compute the dot product between e_j and e_x within the angled brackets.

  Response:
  Eq. (5) refers to a statistical average. We have added a clarification to the text.

- In section "Nematic order determines anisotropy of network permeability," you refer to your Monte Carlo network generation algorithm as a "stochastic network generation algorithm." This is technically correct and accurate, but I would encourage you to replace 'stochastic' with 'Monte Carlo' for the sake of consistency and clarity

  Response:
  We agree and have changed the wording as suggested.

- In section "Nematic order determines anisotropy of network permeability," you refer to Darcy's law, but do not provide a reference. Please provide a reference

  Response:
  We apologize for this oversight and have now added a reference to Darcy's law by Whitaker. The seminal theory paper by Stephen Whitaker presents a theoretical derivation of Darcy's law for porous media; note that the original experimental work

by Henry Darcy (*Les fontaines publiques de la ville de Dijon*) would be more difficult to access for readers.

- For fluid transport simulations, you make the assumption that the pressure drop across the boundary of the cuboid region is uniform (pressure drop along *x*-axis; at *x*=0 all nodes are set to $p_0 + \Delta p$, and all nodes at *x*=*L* nodes are set to p_0). While this is a practical choice, it more than likely does not reflect the true physiology of hepatic sinusoid networks. You should discuss the limitations of this assumption, and how this may impact your predictions on permeability and network resilience.

Response:
Indeed, in the liver lobule, blood flows from the portal vein to the central vein, which is sketched in Fig. 1B. Yet, in the central region of the liver lobule, both mean flow and local nematic alignment direction of the sinusoidal network are approximately directed along a single, common axis. We first provide support for this statement, then discuss its implications for our choice of region-of-interest:
- First, based on previous continuum simulations [e.g., Debbaut et al. 2012], blood flow from portal to central vein is expected to be approximately oriented along the *x*-axis, but to deviate in the vicinity of the large veins.
- Second, in previous work [Morales et al. 2019], we computed the nematic director field of sinusoidal networks (i.e., a field of local directions of nematic alignment of the sinusoid network at different positions). We then found that this director field followed curvilinear paths, similar to potential field lines connecting portal and central vein. Importantly, in the central region of the liver lobule, nematic alignment direction was approximately uniform.

Thus, by choosing a region-of-interest in the central region of the liver lobule, we can avoid the expected curvilinearity of flow patterns to a large extent. Obviously, choosing the size of this region of interest amounts to a compromise between selecting a large sample and restricting the analysis to a region with approximately constant direction of the nematic director field.
Lastly, we emphasize that our goal was not the computation of flow profiles with physiological boundary conditions, but instead the characterization of the transport properties of the network, using network permeability as a size-independent "material property" of this spatial networks. This goal is best achieved by choosing boundary conditions with a pressure difference on opposing sides of the region of interest in the center of the lobule.

We have added the following sentence to the revised manuscript:
We will quantify these [anisotropic transport properties] by an anisotropic permeability, which represents a size-independent "material property" of the spatial network. This will facilitate the comparison to simulated networks, as opposed to flow computations for physiological boundary conditions with influx and outflux at the central and portal vein, respectively. …
The rationale [for pressure boundary conditions at opposite boundaries] is that both the direction of mean flow from previous continuum models of flow [Debbaut2012],

as well as the local nematic direction of the sinusoidal network are approximately uniform in the central region of the liver lobule, parallel to the CV-PV axis. For the chosen region of interest, these directions are thus expected to be perpendicular to region boundaries with the imposed pressure difference.

- In Figure 4A, you should add legend labels for the different colors and line types. These are described in the figure caption, but the figure would be clearer with these labels

  Response:
  In the original submission, we had such labels in Figure 4A, but placed these to the right side of the plot, which might have been confusing.
  We have now re-arranged the labels in this figure panel.

Comments/Suggestions

- In section "Nematic order determines anisotropy of network permeability," you mention that inhomogeneous distribution of flows in the network is not intuitive. Why is this contrary to your intuition? What was your original expectation? There is a deep and expansive literature on the fractal nature of vascular networks in which models have predicted and experiments have validated that the distribution of blood flow becomes more heterogeneous as smaller and smaller blood vessels are observed - see [*Glenny, R. W., Bernard, S. L., & Robertson, H. T. (2000). Pulmonary blood flow remains fractal down to the level of gas exchange. Journal of Applied Physiology, 89(2), 742-748.]*. Moreover, in the final paragraph of your discussion you mention that disordered networks are characterized by an inhomogeneous distribution of flow. There appears to be some disconnect in whether you expect or do not expect flow in a microvascular network to be inhomogeneously distributed.

  Response:
  Our initial expectation was to find a homogeneous distribution of flow in the sinusoidal network. The first reason that led us to this expectation is that all hepatocytes need to be evenly supplied with oxygen and nutrients by the sinusoidal network. The second reason is that the sinusoidal microvasculature in the liver lobule is plexus-like - in contrast to hierarchical networks found e.g. in the lung. In principle, the many loops and cross-connections in the sinusoidal network could homogenize the distribution of flow.

  The result of our analysis is that
  - either the distribution of flow is indeed inhomogeneous, contrary to our initial expectation, or
  - additional, adaptive mechanisms, e.g., adaptive regulation of sinusoid diameter ensure a more homogeneous distribution of flow.

  We now discuss previous work that similarly reported inhomogeneous distribution of flow at small scales in blood networks in the discussion section:

While similar observations had been made previously for hierarchical networks [Yipintsoi et al. 1973, Glenny et al. 2000], we had expected a more homogeneous distribution in the plexus-like sinusoidal network due to its high number of loops, also because hepatocytes require a homogeneous supply with oxygen and nutrients throughout the liver lobule.

We also rephrased the respective sentence on disordered networks in the 'Discussion' section to connect it better to the discussion of experimental networks:
As a benchmark, we compared permeability-at-risk curves of sinusoidal networks to those of both regular lattices, as an example of ordered networks, as well as sub-lattices of such regular lattices, as an example of disordered networks. We find that the regular lattices exhibit higher resilience, whereas disordered networks have similar permeability-at-risk curves as the sinusoidal network. We attribute this lower resilience of disordered lattices to the presence of a small fraction of edges that carry substantially higher currents. Indeed, we observed an inhomogeneous distribution of flows also in the disordered network, but not in the regular networks.

- In the discussion you reference how physiological regulation (ex: shear dependent vasodilation, transient clogging of the network by RBCs) may play a role in homogenizing the distribution of blood flow in a network. Have you considered trying to implement one or more of these physiological controls in your flow simulations? Tim Secomb and Axel Pries are dominant names in this field (representative example – [Pries, A. R., Secomb, T. W., Gaehtgens, P., & Gross, J. F. (1990). Blood flow in microvascular networks. Experiments and simulation. *Circulation research*, *67*(4), 826-834.]) where they model and make experimental measurements of blood flow in mesenteric networks accounting for a number of regulatory mechanisms. I don't think it is fair for me to ask you to add these kinds of simulations to this paper, but you should discuss how physiological regulation may impact your predictions on permeability and network resilience. If the flow distribution is more homogenous, perhaps the removal of a high flow edge would be less deleterious compared to your simulated inhomogeneous flow distributions.

Response:
We did cite the relevant work by Pries et al. before, yet agree that we can address the adaption mechanisms proposed therein in greater detail.
We now write
As a second effect, at branch points of the network, the daughter branch with the higher flow will receive on average a fraction of red blood cells that is higher than the proportional share [Pries et al. 1989]. This so-called phase-separation or network Fahraeus effect could result in an even more inhomogeneous distribution of hematocrit.

Moreover, we formulated a minimal model of network adaptation and added a new SOM figure, see SOM text. There, we introduce adaptive weights for individual edges; these weights are adapted dynamically according to whether the current through an individual edge is above or below a fixed threshold. We find that such a local adaptation rule can indeed homogenize the distribution of currents in the network, see

new Figure S4. However, this minimal model cannot alleviate the structural vulnerability to the removal of high-current edges.
It will indeed an interesting research direction to explore, which local or global adaptation rules render plexus-like transport networks more robust with respect to removal of high-current edges. However, we feel that such an endeavor would go beyond the scope of the current manuscript.

- Microvascular networks are not static structures. New vessels are grown through angiogenesis, and older vessels are pruned in a dynamic fashion. This dynamic ability of vascular networks to remodel themselves certainly plays a role in their ability to respond to maintain function in response to all manner of stressors (such as the loss of a vessel segment/edge). You should discuss how vascular network remodeling contributes to their resilience to vessel segment/edge loss. In my opinion, the difficult thing to predict here is the interaction between the time course in which an injury that causes vessel/edge loss, and the time course it takes for the vascular network to remodel.

Response.
Adaptive remodeling of sinusoidal networks is important and is expected to occur on a time-scale of weeks. In fact, the observed statistics of these networks could reflect the steady-state of dynamic adaptation with yet unknown set-points.
In light of the remarkable regenerative capabilities of liver tissue, we expected that any damage of the sinusoidal network should be compensated on a time-scale of several days or weeks.
The resilience properties addressed in our manuscript are relevant on shorter time-scales, between seconds for transient clogging to days for damaged vessels.
In the revised manuscript, we have added a corresponding sentence in the Introduction:
The liver exhibits remarkable regeneration capabilities. After local damage, sinusoidal networks are expected to self-repair on a time-scale of days and weeks. Yet, on shorter time-scales, the sinusoidal network must cope with any transient reduction in network permeability. By how much the permeability of the sinusoidal network will be reduced after a perturbation is not known.

Have all data underlying the figures and results presented in the manuscript been provided?
Large-scale datasets should be made available via a public repository as described in the *PLOS Computational Biology* data availability policy, and numerical data that underlies graphs or summary statistics should be provided in spreadsheet form as supporting information.

Reviewer #1: Yes

Reviewer #2: None

Response: We took up the suggestion of review #2 and have now included data sets of sinusoidal networks as Supplemental Data File.

New supplemental data file:
sinusoidal_networks_liver_tissue.zip
