# Supplementary material for: Resilience of three-dimensional sinusoidal networks in liver tissue
Source: PLoS Comput Biol. 2020 Jun 29;16(6):e1007965. doi: 10.1371/journal.pcbi.1007965 (PMC7351228; doi:10.1371/journal.pcbi.1007965)
Supplement: Attachment — Submitted filename: karschau_network_resilience_2nd_revision.pdf [file pcbi.1007965.s006.pdf]

TECHNISCHE UNIVERSITÄT DRESDEN

Technische Universität Dresden, 01062 Dresden

Editorial Office
PLoS Computational Biology

PD Dr. rer. nat. habil. Benjamin M. Friedrich
Biological Algorithms Group

Tel: +49 351 463 42641
E-Mail: benjamin.m.friedrich@tu-dresden.de
URL: https://www.cfaed.tu-dresden.de/friedrich-home

PCOMPBIOL-D-19-02210 / Resubmission
Karschau et al. Resilience of three-dimensional sinusoidal networks in liver tissue

Dear Prof. Daniel A Beard,
Dear Editorial Staff,

May 16, 2020

We feel honored by the positive recommendation of both reviewers.
Hereby, we resubmit a second revision of our manuscript that addresses the last open critique of reviewer #2 as well as his (optional) clarity suggestions.

We summarize the changes to our manuscript (point-by-point answers to all comments of the referees are included further below):

- We now state that results for the adaptive network are insensitive to the choice of the set point I^*, as long as I^* falls in the range of typical currents of the unperturbed network; for Fig. S4 we had chosen I^* as the 95% percentile of the currents in the unperturbed network.
- We added a sentence to clarify that the normalized permeability K_{xx} is indeed independent of the single-edge resistance parameter κ_0.

We are confident that this second revision meets the high standards of PLoS Computational Biology.

On behalf of all authors,
Yours sincerely,
Benjamin M Friedrich

Visiting Address:
Barkhausen-Bau
Helmholtzstr. 18
Room II60A (D-wing)
01069 Dresden
Germany

www.tu-dresden.de

DRESDEN concept
Exzellenz aus Wissenschaft und Kultur

We thank both referees for their positive evaluation of our work.

Answer to Referee #1

I am satisfied with the changes made by the authors and recommend publication.

If the authors would like to add the new supplemental figures they mentioned at a later stage that could be good, but I feel that the paper is sufficiently well supported at this time.

Response.
We decided to resubmit the manuscript without those supplemental figures (i.e., simulated networks matching other experimental data sets of sinusoidal networks) to avoid delays.

As of today, it is not clear when physical access to our university buildings will be allowed again, which would be a practical prerequisite to re-run simulations. From preliminary work we can assess that results are very similar to those shown in Figure 2 and 3.

Reviewer #2

Overall, the authors have done an excellent job with this resubmission. I am particularly impressed with the addition of the minimal model of an adaptive network, and the additional details on Pareto optimality brought up by the other reviewer. I only have one major critique and have identified a few typos and clarity suggestions. Treat my typo and clarity suggestions as optional. This is an interesting and well-done study and I am looking forward to seeing it published.

Critiques

In the "Minimal model for adaptive edge weights" section, you describe a model that regulates edge permeability in a way that is dependent upon edge current going below a set point (I*). How was this set point determined? Are the results sensitive to the choice of set point, I*?

Response:

Regarding the critique, we have added the following sentence:

> These results are insensitive to the choice of set point I^*, provided I^* falls in the range of typical currents of the original network."

The value chosen for Fig. S4 corresponds to the 95% percentile of the distribution of currents in the unperturbed network; we have updated the caption of figure S4 to include this information.

We now also follow the suggestion of reviewer #2 and write the right-hand side of the differential equation as a piecewise linear function.

Finally, we have added the following sentence:

Note that the normalized permeability K_{xx} shown in Fig. S4 is independent of κ_0.

Typos and clarity suggestions

In the 7th paragraph of the introduction, you say "Permeabilities allow to efficiently … ", and I think meant to say "Permeabilities allow us to efficiently …"

In the 4th paragraph of the "A network generation algorithm for spatial networks" section, you say "which is in particular robust …", and I think meant to say "which is particularly robust …"

In the first paragraph of the "Discussion and Outlook" section, you say "We drew advantage …" and I think meant to say "We took advantage …"

In the 6th paragraph of the "Discussion and Outlook" section, you say "may facilitate a more homogenous distribution instead." – I think you can delete the word "instead" from this sentence

In the "Permeability-at-risk if low-current edges removed" section, I think the section could be re-named "Permeability-at-risk if low-current edges are removed"

In the "Permeability-at-risk if low-current edges removed" section, you say "However, if together …" and I think you meant to say "However, if performed together"

In equation S3, it appears that kappa_0 cancels out. Is this case?

Your reasoning behind the use of kappa/kappa_0 as a criterion to constrain the change in permeability by a factor of 4 is sound, but perhaps you can express equations S3 as a piecewise function where dkappa_j/dt = -rho kappa (I* - |I_j|) if 0.25 < kappa/kappa_0 < 4, and dkappa_j/dt = 0 otherwise

Response:

We have addressed all clarity suggestions, with the exception of 'allowed us to efficiently compute', since we refer to work of colleagues in this sentence, not our own work.
