# Supplementary material for: Resilience of three-dimensional sinusoidal networks in liver tissue
Source: PLoS Comput Biol. 2020 Jun 29;16(6):e1007965. doi: 10.1371/journal.pcbi.1007965 (PMC7351228; doi:10.1371/journal.pcbi.1007965)
Supplement: Attachment — Submitted filename: My_review.docx [file pcbi.1007965.s004.docx]

**Review of “Resilience of three-dimensional sinusoidal networks in liver tissue”**

**Summary**

This study is motivated by the question “can 3D microvascular networks work if individual links fail?” The authors first quantify the statistical geometry of high-resolution images of mouse hepatic sinusoidal networks. They operate from a graph theory perspective to define the bifurcations as nodes and the blood vessels as edges. They use a Monte-Carlo algorithm to generate synthetic networks which reproduce the statistical features of mouse hepatic sinusoidal networks. Node degree and edge length are the optimized quantities. The authors also quantify the bulk direction network edges (blood vessels) are oriented by a nematic order parameter which is also included in their multi-objective cost function for Monte-Carlo network generation. A minimal model of blood flow is used to predict the transport properties of the network. The critical assumptions made are that blood vessels have a constant resistance per unit length, and that there is a uniform pressure drop across the simulated cuboid region boundary (pressure drop along x-axis; at x=0 all nodes are set to p0 + delta P, and all nodes at x=L nodes are set to p0). Normalized permeability of the network is computed via Darcy’s law. Network resilience is quantified by a new metric called “permeability-at-risk,” where normalized permeability is plotted as a function of the fraction of removed edges. Two scenarios are considered for the removal of edges: (i) removal of high current edges; and (ii) removal of random edges. Simulations of these two scenarios are used to address the original question – can microvascular networks work if individual links fail?

**Major Findings**

- The sinusoidal network is a subgraph of the Delaunay graph (of the bifurcation nodes). Implies that sinusoidal networks reflect design requirements in which every hepatocyte in the tissue is in contact with the vascular network
- The sinusoidal network contains the minimum spanning tree. Implies that sinusoidal network design/growth minimizes path lengths throughout the network
- Nematic order determines the anisotropy of network permeability.
- Removing high flow edges is more detrimental compared to random removal of edges. At a given fraction of edges removed from the network, removing high flow edges makes it more likely for the permeability (in the direction of the pressure drop) to collapse to zero. In other words, low flow edges enable network resilience.

**Critiques**

- Nematic alignment/order needs to be clearly defined at the beginning of the “Nematic order determines anisotropy of network permeability” section. A quick google search of the term gives a lot of information on liquid crystals in LCD TV displays. By context I was eventually able to infer what it was measuring, but it was not immediately intuitive.
- In equation (5), you should clarify what the angled brackets indicate. Is it an inner product, or a statistical average? My guess is that it’s an average, as you compute the dot product between e_j and e_x within the angled brackets.
- In section “Nematic order determines anisotropy of network permeability,” you refer to your Monte Carlo network generation algorithm as a “stochastic network generation algorithm.” This is technically correct and accurate, but I would encourage you to replace ‘stochastic’ with ‘Monte Carlo’ for the sake of consistency and clarity
- In section “Nematic order determines anisotropy of network permeability,” you refer to Darcy’s law, but do not provide a reference. Please provide a reference
- For fluid transport simulations, you make the assumption that the pressure drop across the boundary of the cuboid region is uniform (pressure drop along x-axis; at x=0 all nodes are set to p0 + delta P, and all nodes at x=L nodes are set to p0). While this is a practical choice, it more than likely does not reflect the true physiology of hepatic sinusoid networks. You should discuss the limitations of this assumption, and how this may impact your predictions on permeability and network resilience.
- In Figure 4A, you should add legend labels for the different colors and line types. These are described in the figure caption, but the figure would be clearer with these labels

**Comments/Suggestions**

- In section “Nematic order determines anisotropy of network permeability,” you mention that inhomogeneous distribution of flows in the network is not intuitive. Why is this contrary to your intuition? What was your original expectation? There is a deep and expansive literature on the fractal nature of vascular networks in which models have predicted and experiments have validated that the distribution of blood flow becomes more heterogenous as smaller and smaller blood vessels are observed - see [*Glenny, R. W., Bernard, S. L., & Robertson, H. T. (2000). Pulmonary blood flow remains fractal down to the level of gas exchange. Journal of Applied Physiology, 89(2), 742-748.].*  Moreover, in the final paragraph of your discussion you mention that disordered networks are characterized by an inhomogeneous distribution of flow. There appears to be some disconnect in whether you expect or do not expect flow in a microvascular network to be inhomogeneously distributed.
- In the discussion you reference how physiological regulation (ex: shear dependent vasodilation, transient clogging of the network by RBCs) may play a role in homogenizing the distribution of blood flow in a network. Have you considered trying to implement one or more of these physiological controls in your flow simulations? Tim Secomb and Axel Pries are dominant names in this field (representative example – [Pries, A. R., Secomb, T. W., Gaehtgens, P., & Gross, J. F. (1990). Blood flow in microvascular networks. Experiments and simulation. *Circulation research*, *67*(4), 826-834.]) where they model and make experimental measurements of blood flow in mesenteric networks accounting for a number of regulatory mechanisms. I don’t think it is fair for me to ask you to add these kinds of simulations to this paper, but you should discuss how physiological regulation may impact your predictions on permeability and network resilience. If the flow distribution is more homogenous, perhaps the removal of a high flow edge would be less deleterious compared to your simulated inhomogeneous flow distributions.
- Microvascular networks are not static structures. New vessels are grown through angiogenesis, and older vessels are pruned in a dynamic fashion. This dynamic ability of vascular networks to remodel themselves certainly plays a role in their ability to respond to maintain function in response to all manner of stressors (such as the loss of a vessel segment/edge). You should discuss how vascular network remodeling contributes to their resilience to vessel segment/edge loss. In my opinion, the difficult thing to predict here is the interaction between the time course in which an injury that causes vessel/edge loss, and the time course it takes for the vascular network to remodel.
